# Hazard-Rate Analysis and Patterns of Recurrence in Early Stage Melanoma: Moving towards a Rationally Designed Surveillance Strategy

**DOI:** 10.1371/journal.pone.0057665

**Published:** 2013-03-13

**Authors:** April K. S. Salama, Nicole de Rosa, Randall P. Scheri, Scott K. Pruitt, James E. Herndon, Jennifer Marcello, Douglas S. Tyler, Amy P. Abernethy

**Affiliations:** 1 Department of Medicine, Duke University Medical Center, Durham, North Carolina, United States of America; 2 Duke Cancer Institute, Duke University Medical Center, Durham, North Carolina, United States of America; 3 Department of Surgery, Duke University Medical Center, Durham, North Carolina, United States of America; 4 Department of Biostatistics and Bioinformatics, Duke University Medical Center, Durham, North Carolina, United States of America; University of Tennessee, United States of America

## Abstract

**Background:**

While curable at early stages, few treatment options exist for advanced melanoma. Currently, no consensus exists regarding the optimal surveillance strategy for patients after resection. The objectives of this study were to identify patterns of metastatic recurrence, to determine the influence of metastatic site on survival, and to identify high-risk periods for recurrence.

**Methods:**

A retrospective review of the Duke Melanoma Database from 1970 to 2004 was conducted that focused on patients who were initially diagnosed without metastatic disease. The time to first recurrence was computed from the date of diagnosis, and the associated hazard function was examined to determine the peak risk period of recurrence. Metastatic sites were coded by the American Joint Committee on Cancer (AJCC) system including local skin, distant skin and nodes (M1a), lung (M1b), and other distant (M1c).

**Results:**

Of 11,615 patients initially diagnosed without metastatic disease, 4616 (40%) had at least one recurrence. Overall the risk of initial recurrence peaked at 12 months. The risk of initial recurrence at the local skin, distant skin, and nodes peaked at 8 months, and the risk at lung and other distant sites peaked at 24 months. Patients with a cutaneous or nodal recurrence had improved survival compared to other recurrence types.

**Conclusions:**

The risk of developing recurrent melanoma peaked at one year, and the site of first recurrence had a significant impact on survival. Defining the timing and expected patterns of recurrence will be important in creating an optimized surveillance strategy for this patient population.

## Introduction

Melanoma is one of the few cancers whose incidence continues to rise, and in 2012, an estimated 76,250 new cases will be diagnosed in the United States [1]. Fortunately, the majority of these patients are diagnosed with localized disease and are considered cured. Despite this, approximately 20–30% of early stage melanoma patients develop a recurrence during their lifetime, with much higher rates seen in patients with regionally advanced disease [2]. The optimal timing and modality of surveillance after initial diagnosis and treatment is not known. Historically, routine imaging studies have not been shown to improve outcomes, and additional concerns have been raised regarding the risks of radiation exposure, the high false positivity rate and cost effectiveness [3], [4], [5], [6]. Additionally, recent data suggests that frequent exams over an extended time period may be of limited benefit [7], [8]. Currently, clinical practice varies widely and there is no consensus regarding the type, timing, or duration of radiologic or clinical evaluations. Timing of imaging surveillance is often guided by the data collection processes used in clinical trials, practical aspects of clinical care, experience in other cancers, and anecdote. Ideally, surveillance patterns would be matched to objective observations about metastatic patterns of spread in melanoma and risk for subsequent relapse. The lack of an improvement in outcomes with screening in historical series is likely influenced by the limited availability of systemic agents to alter the course of advanced disease, a landscape that has changed markedly over the past few years. The objective of this study was to determine high-risk periods of recurrence by metastatic site as a first step to designing practical surveillance care for patients with melanoma. For the over 800,000 people currently living in the United States with a previous diagnosis of melanoma, a rationally designed surveillance strategy could allow for improved disease outcomes, time savings, and a reduction in wasteful health resource utilization [9].

## Methods

### Population

All patients with melanoma at Duke University Medical Center were entered into a prospectively maintained computerized, single-institution registry database from 1970 to 2004. A retrospective review was conducted. All patients had histologically confirmed melanoma. Patient data including clinical characteristics, pathologic findings, date and status at last follow-up were entered prospectively into the database by trained data managers. Approval for this study was obtained from the Duke University Health System Institutional Review Board, and a waiver for written consent was granted. Because this study was a retrospective database review, it was felt to only represent a minimal risk of loss of confidentiality to any included patient. Precautions were taken to further minimize this risk, and all data were analyzed anonymously. Only the principal investigator and key personnel had access to the identified master file, which was stored in a secured location.

The Duke Melanoma Database is comprised of data from 14,029 patients. Exclusion criteria for this study included patients who presented with metastatic disease at diagnosis, who had a recorded recurrence within 30 days of initial diagnosis, or who were followed for less than 30 days; after these exclusions there were 11,615 patients included in the analysis.

### Classification of recurrence

The first 3 sites of recurrence were summarized for each patient. Metastatic sites were categorized into 4 groups corresponding with American Joint Cancer Committee staging criteria: 1) local skin; 2) distant skin and lymph nodes including intransit nodes; 3) lung; and, 4) other distant sites which included adrenal glands, bone, central nervous system, eye, gastrointestinal, liver, other, and death due to metastases but not otherwise specified. If multiple recurrences occurred within 30 days of each other (in either direction) then recurrences were combined and counted as one event; the event was assigned the date of the earliest recurrence and the site was classified according to that site with the worst expected prognosis based on current staging criteria such that non-pulmonary visceral disease represented the poorest prognositc group, followed by patients with pulmonary metastases, distant skin and nodes, and local skin recurrence only.

### Statistical methods

Patient characteristics and the pattern of metastatic spread from the first to third recurrence were summarized using basic descriptive statistics. Contingency tables were used to compare patient characteristics (sex, Clark's level, ulceration, Breslow thickness, primary site, and histology) and type of lymph node procedure between patients with and without recurrences. The mean age by recurrence status was computed. Kaplan-Meier survival estimates and plots were produced to summarize time from diagnosis to death by recurrence status (no recurrence vs. at least one recurrence). Among patients who had at least one recurrence, survival from time of first recurrence was summarized by site of first recurrence.

The period of highest risk for recurrence was evaluated by plotting the hazard function for time to 1^st^, 2^nd^ and 3^rd^ recurrence and noting the maximum hazard. The hazard function was estimated from the Kaplan-Meier survival function using the Epanechnikov Kernal smoothing method with a bandwidth of 8 and 101 grid points. Time to first recurrence was calculated from initial diagnosis and patients who did not have any recurrences were censored at their time of death or date last contact. Time to second recurrence was calculated for all patients who had at least one recurrence from date of first recurrence to date of 2^nd^ recurrence or date of death/last contact if censored. Likewise, time to third recurrence was calculated among patients with at least 2 recurrences as time from 2^nd^ recurrence to time to 3^rd^ recurrence or date of death/last contact if censored. Since we defined a new recurrence to be at least 30 days from any previous recurrence, any patient who died or was otherwise lost to follow-up less than 1 month after their last recurrence was considered not to be at risk of recurrence and was excluded from calculation of the hazard function.

The hazard of a first recurrence in local skin, distant skin/nodes, lung, or other distant metastasis was estimated for each site separately and then plotted together for comparison. The definition for time to first recurrence remained the same when estimating each of the 4 hazard functions, however patients whose recurrence was not at the site being estimated were censored at the time of their first recurrence.

All data manipulation and analysis was performed with SAS v9.2 (Cary, NC).

## Results

From 1970 to 2004, 14,029 patients with melanoma were treated at Duke Univerisity Medical Center. Patients who presented with metastatic disease at diagnosis, who had a recorded recurrence within 30 days of initial diagnosis, or who were followed for less than 30 days were excluded from this analysis. Of the 11,615 eligible patients, 4,616 (40%) had at least one recurrence during long-term follow-up. The clinicopathologic characteristics of these patients are shown in Table 1.

**Table 1:** Patient characteristics.

	No recurrences		At least one recurrence		Total	
**Sex (%)**						
Male	3592	(51.3)	2696	(58.4)	6288	(54.1)
Female	3407	(48.7)	1920	(41.6)	5327	(45.9)
**Age at Diagnosis**						
Mean (SD)	49 (15.1)		48 (14.9)		48.6 (15.0)	
**Thickness of Invasion mm (%)**						
≤1.00	2033	(29.0)	665	(14.4)	2698	(23.2)
1.01–2.0	2256	(32.2)	1237	(26.8)	3493	(30.1)
2.01–4.0	1076	(15.4)	1050	(22.7)	2126	(18.3)
>4.0	387	(5.5)	583	(12.6)	970	(8.4)
Missing/not recorded	1247	(17.8)	1081	(23.4)	2328	(20.0)
**Clarks level of primary (%)**						
Level I/In situ	281	(4.0)	58	(1.3)	339	(2.9)
Level II	691	(9.9)	205	(4.4)	896	(7.7)
Level III	2499	(35.7)	1322	(28.6)	3821	(32.9)
Level IV	2634	(37.6)	2015	(43.7)	4649	(40.0)
Level V	211	(3.0)	307	(6.7)	518	(4.5)
Unknown level	173	(2.5)	523	(11.3)	696	(6.0)
Does not apply	510	(7.3)	186	(4.0)	696	(6.0)
**Ulceration (%)**						
No	5496	(78.5)	2804	(60.7)	8300	(71.5)
Yes	712	(10.2)	1035	(22.4)	1747	(15.0)
Missing/not recorded	791	(11.3)	777	(16.8)	1568	(13.5)
**Site of primary melanoma (%)**						
Trunk	2586	(36.9)	1987	(43.0)	4573	(39.4)
Upper Extremity	1270	(18.1)	554	(12.0)	1824	(15.7)
Lower Extremity	1415	(20.2)	945	(20.5)	2360	(20.3)
Head/Neck	1176	(16.8)	885	(19.2)	2061	(17.7)
Eye	516	(7.4)	153	(3.3)	669	(5.8)
Other	36	(0.5)	91	(2.0)	127	(1.1)
Missing/not recorded	0	(0)	1	(0)	1	(0)

### Hazard Risk of Recurrence

**Figure 1:**
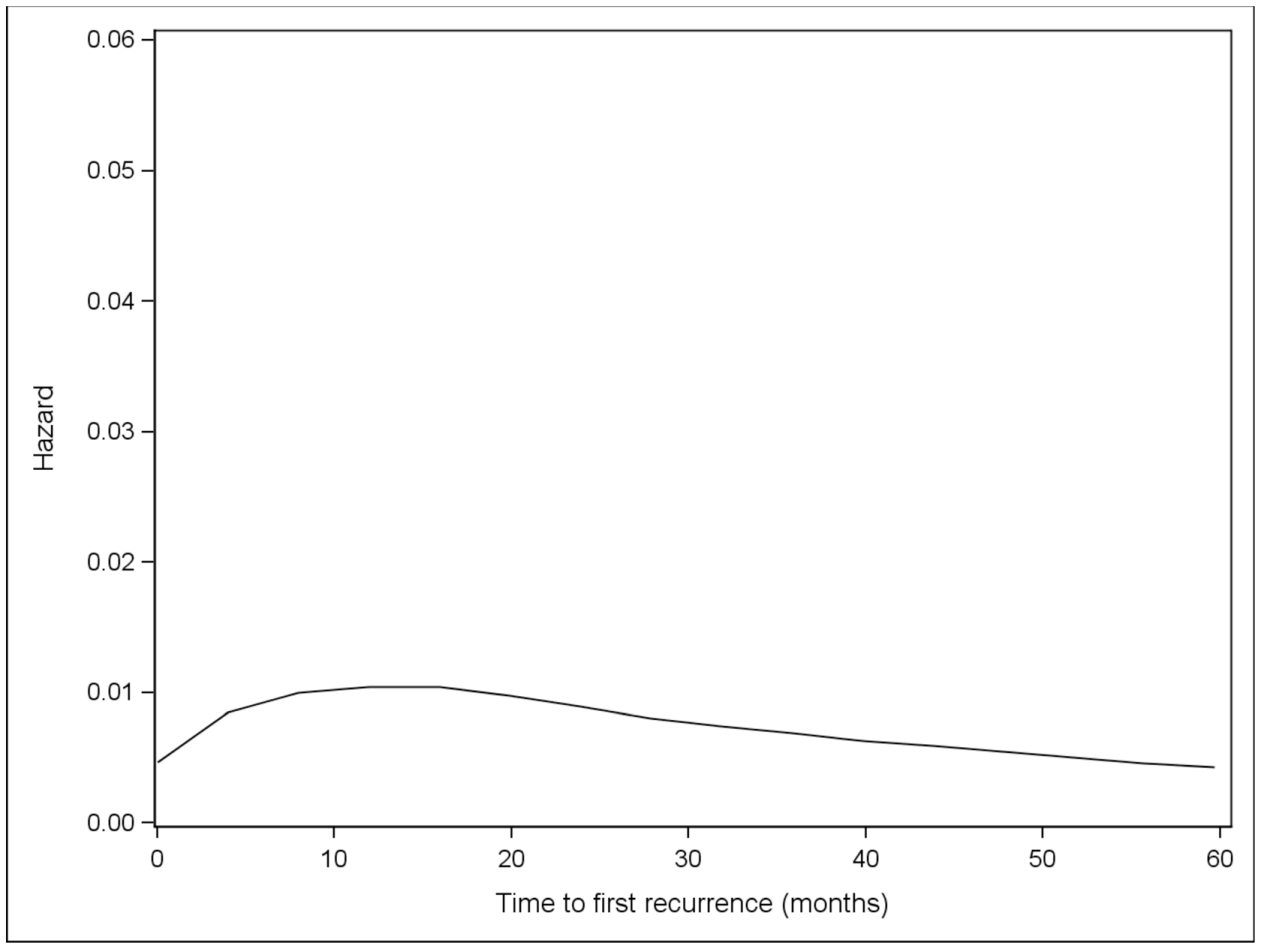
Hazard plot for first recurrence among all patients.

**Figure 2:**
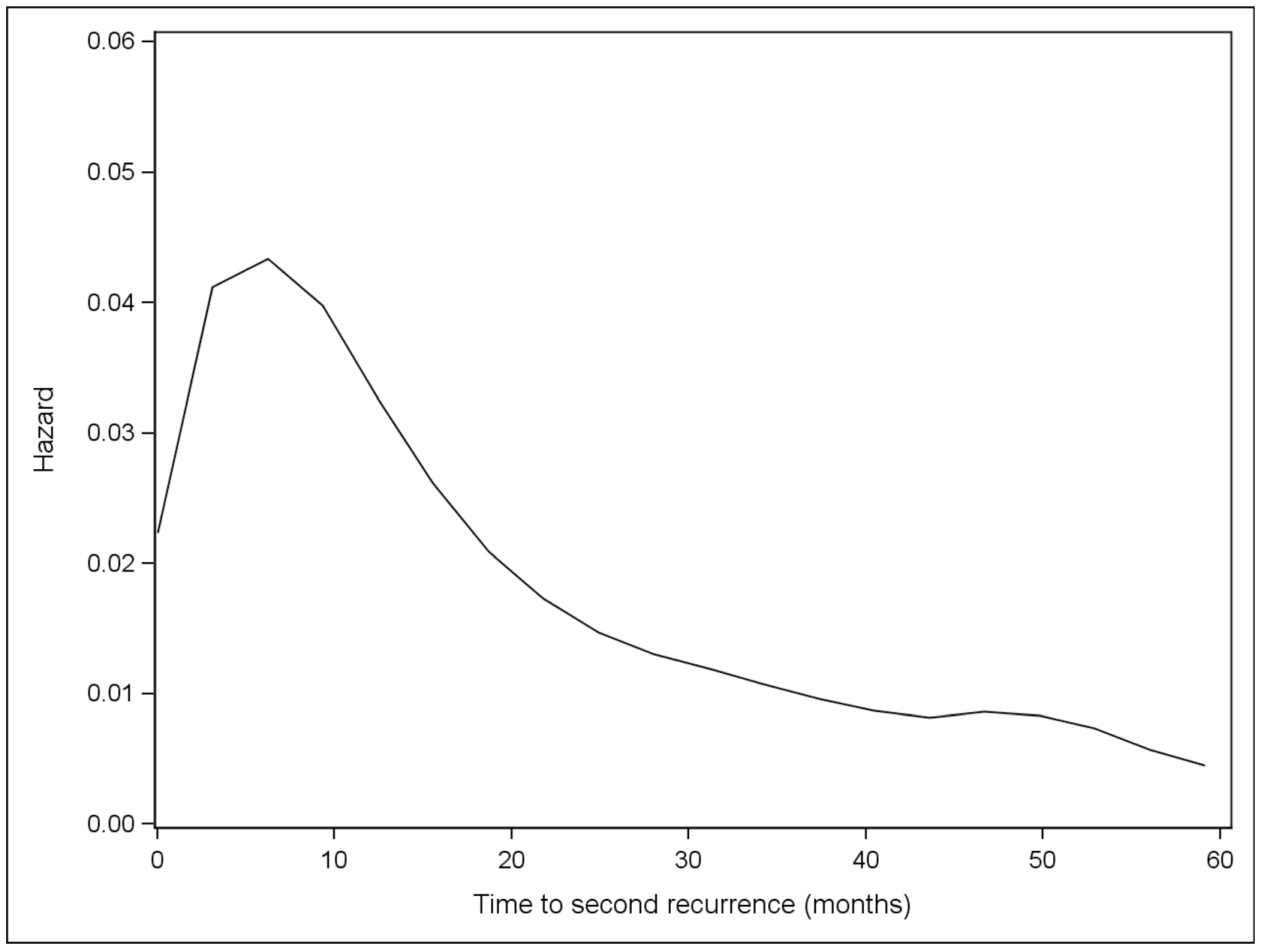
Hazard plot for 2^nd^ recurrence among all patients with at least one recurrence.

**Figure 3:**
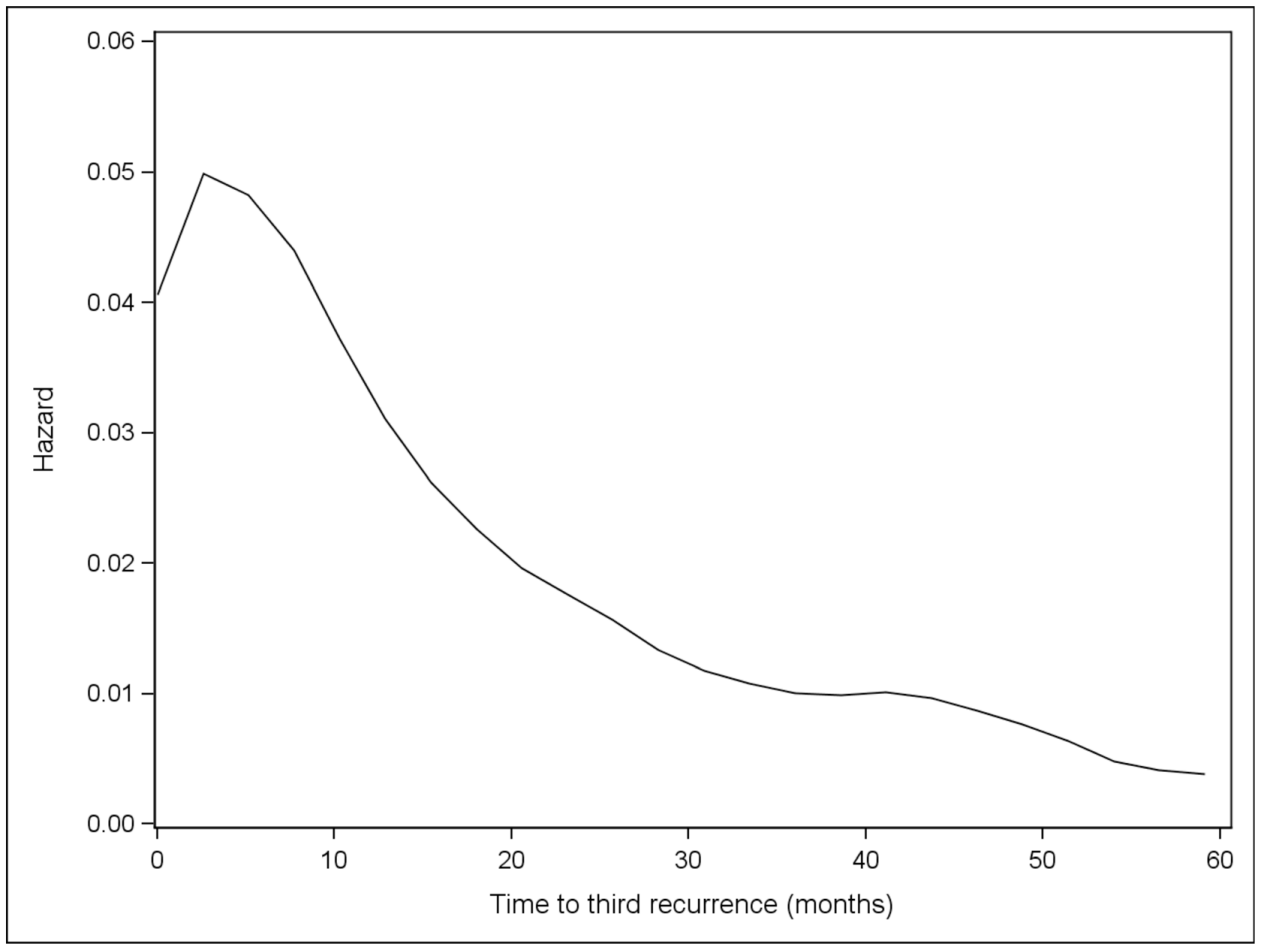
Hazard plot for 3^rd^ recurrence among all patients with at least two recurrences.

Overall, the risk of initial recurrence peaked at 12 months (Figure 1). The risk of initial recurrence at local skin, distant skin and nodes occurred early and peaked at 8 months while the risk of initial recurrence at lung and other distant sites occurred later and peaked at 24 months. After the initial recurrence, subsequent metastases appeared at progessively shorter intervals, with the time to development of second and third metastases peaking at 6.2 and 2.6 months, respectively (Figures 2 and 3). Although the risk of recurrence decreased over time, it did not reach zero.

### Patterns of Metastatic Disease

The most common site of initial recurrence was distant skin or nodes (59%). Of these patients, 91% experienced a nodal recurrence. One quarter of patients in this study had no lymph node assessment documented, while 42% of these patients underwent a completion lymph node dissection, 1.7% had a sentinel lymph node biopsy, and 31% had some type of other lymph node procedure, as listed in Table 2. The second most common site of metastasis was other distant metastases (16.5%), followed by local skin (16.1%), and lung (8.4%) (Table 3). In order to determine if the initial site of metastasis was predictive of future sites of recurrence, sites of second and third recurrences were analyzed. Patterns of spread varied widely across the melanoma population, and an appreciable pattern was not identified. Table 4 lists the incidence of second recurrence by site of first recurrence.

**Table 2:** Summary of lymph node procedures performed.

	no recurrence		1+ recurrences (any site)		All	
	N	(%)	N	(%)	N	(%)
Had sentinel lymph node biopsy	255	(3.6)	79	(1.7)	334	(2.9)
Had full node dissection (no SLN)	1119	(16)	1917	(42)	3036	(26)
Had other lymph node procedure	152	(2.2)	1446	(31)	1598	(14)
No lymph node procedures recorded	5473	(78)	1174	(25)	6647	(57)

**Table 3:** Incidence of first recurrence by site.

	N	%
**Local skin**	742	16.1
**Distant skin + nodes**	2725	59.0
Nodes: 2487(91.3%)		
Distant Skin: 196(7.2%)		
Both: 42(1.5%)		
**Lung**	386	8.4
**Other distant metastases**	763	16.5
**Total**	4616	100.0

**Table 4:** Incidence of second recurrence by site of first recurrence.

Site 1		Site 2	N pts	
Local skin	None			200
	Local skin			176
	Distant skin + nodes			225
	Lung			65
	Other distant metastases			76
Distant skin + nodes	None			1089
	Local skin			163
	Distant skin + nodes			685
	Lung			225
	Other distant metastases			563
Lung	None			248
	Local skin			9
	Distant skin + nodes			23
	Lung			17
	Other distant metastases			89
Other distant metastases	None			659
	Local skin			2
	Distant skin + nodes			21
	Lung			17
	Other distant metastases			64

### Survival

**Figure 4:**
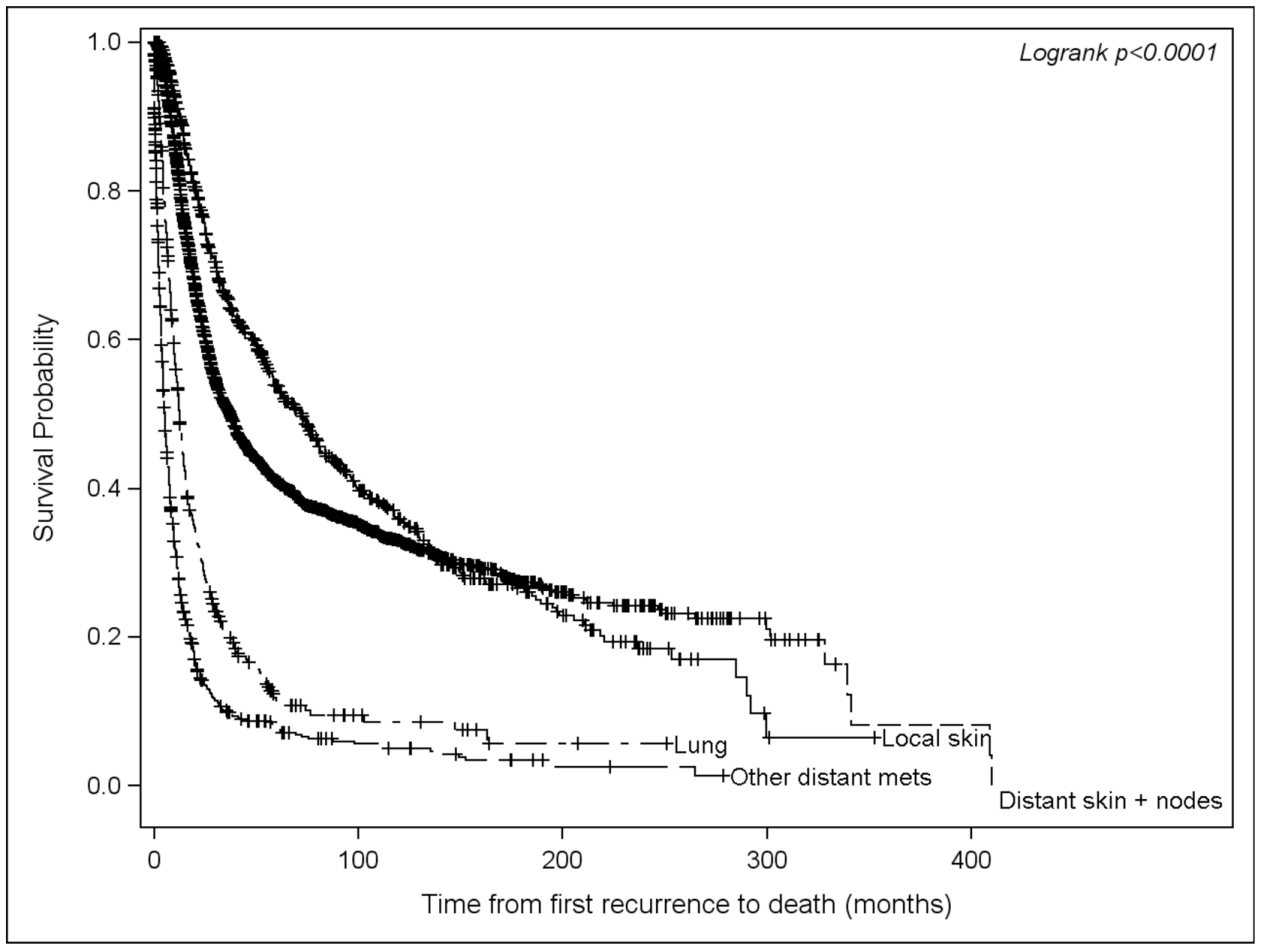
Kaplan-Meier survival from first recurrence by site.

The median 5 and 10 year survival rates from the time of diagnosis were 92.5% (95% CI 91.7–93.2) and 84.3% (83.0–85.4) for patients without a recurrence, compared to 59.8% (58.3–61.2) and 38.9% (37.3–40.5) for patients whose disease had recurred. As expected, there was an association between survival and the initial site of recurrence. Among those who recurred, patients with an initial recurrence in the local skin had the best median survival from the time of recurrence (72 months), followed by distant skin and nodes (37 months), lung (12 months), and other distant metastasis (5 months; Figure 4).

## Discussion

Limited data is available regarding the optimal surveillance strategy for patients diagnosed with early stage melanoma. With over 11,000 patients, this study represents one of the largest cohorts reported to our knowledge. By using a hazard rate analysis, we sought to identify the highest risk period for recurrence in order to define a window where patients are most likely to benefit from surveillance.

In this study, we found that the risk of initial recurrence peaked at 12 months, which is consistent with current data showing that most metastases develop within the first few years after initial diagnosis [7], [8]. The risk of recurrence at local skin, distant skin and nodes occurred early and peaked at 8 months, while distant metastasis to lung and other sites occurred later and peaked at 24 months. The risk of recurrence never returned to zero, as it is well documented that late recurrences can occur in a small percentage of patients, especially those with early stage disease [10], [11], [12], [13], [14], [15], [16]. The pattern of metastatic disease, in which most patients presented with a distant cutaneous or nodal recurrence, is similar to other reported series, and may be reflective of the fact that many patients were diagnosed prior to the routine use of sentinel lymph node biopsies [17], [18]. Subsequent analyses in patients who have undergone a sentinel lymph node biopsy suggest that there is a lower proportion of regional nodal recurrences [19], [20], [21], [22].

Furthermore, our data shows that once a metastatic lesion is identified, the remission period sequentially shortens with the risk of a second recurrence peakng at 6 months, and that of a third recurrence at 2.6 months. Survival, as expected, was also site dependent, with patients who initially developed metastases to the skin or lymph nodes having a longer median survival compared to patients who presented with metastases to visceral organs.

The driving force behind any surveillance strategy in oncology is that detecting a recurrence before a patient becomes symptomatic should have a favorable impact on survival. While case series have suggested that recurrences that are amenable to a surgical intervention (i.e., detected earlier) are associated with improved survival, this principle has not held true for most patients with melanoma [23]. The majority of recurrences are detected by patients themselves, and no imaging modality has provided a clear survival benefit [24], [25]. An analysis by Turner et al showed that a less intensive monitoring schedule would likely only result in a small delay in the time to detect a recurrence, yet has the potential to save thousands of clinic visits over a 10 year course of follow up [7]. Despite the data, the desire to detect (and sometimes the fear of missing) these early recurrences has provided a rationale to adopt some type of surveillance strategy. With the landscape of systemic therapeutic options for advanced melanoma rapidly evolving, patients may stand to benefit from early treatment when they are asymptomatic, highlighting the importance of developing a structured surveillance program that maximizes the potential advantages of early detection without placing an undue burden on patients or the healthcare system.

This study is limited by its retrospective design, and the lack of routine sentinel lymph node biopsies during the time period may influence timing and pattern of recurrence. Additionally, information regarding subsequent therapy and other patient related factors was not possible to identify in this retrospectively analyzed dataset. Nevertheless, this is a large cohort treated at a single institution and it serves to provide an initial framework for analyzing the pattern and timing of disease recurrence- a critical step to the development of any surveillance protocol.

In the absence of definitive data, opinions vary widely and currently no uniform standard exists regarding the appropriate follow up interval, use of imaging modalities, or optimal duration of follow up. The primary site of recurrence was not predictive of a pattern regarding the development of subsequent recurrences, suggesting that it may be difficult to tailor a surveillance strategy to an individual patient and that a standardized approach should be used for all patients once they recur. The initial site of metastasis directly correlated with prognosis in our population, consistent with the current AJCC staging system. Additionally, our study showed that recurrences tended to peak within the first 1–2 years after diagnosis, suggesting that intensive follow up beyond this period may be of lower yield.

Prospective surveillance protocols, designed to assess both the optimal follow-up interval and use of imaging modalities will be critical in order to move forward. Based on our data, structuring a program tailored to risk of recurrence based on the time since diagnosis is one approach for future work. A proposed strategy for surveillance might be to combine routine physical exam with more sensitive radiologic modalities, eg CT or PET/CT, for patients felt to be at high risk of recurrence based on current staging and prognostic information during the first year after diagnosis, as this was the highest risk period for in our overall population. Imaging and clinical exams could be performed at 3–6 month intervals depending on initial risk, and the follow up interval could subsequently be extended in the second and third years. With regards to specific subsets of patients, in this study, only a minority of patients underwent a sentinel lymph node biopsy. In the current era, sentinel lymph node assessment is considered at our institution for most patients with primaries >0.75 mm or thinner lesions with adverse features such as ulceration or an elevated mitotic rate. For patients who meet these criteria, but elect not to proceed with lymph node assessment, focused sonographic imaging and clinical exam of the lymph node basin may be a reasonable option, as a high proportion of nodal recurrences were seen in this study. The Multicenter Selective Lymphadenectomy Trial II (MSLT-2; NCT00297895), in which patients with a positive sentinel node are randomized to receive either serial ultrasound and clinical exams or completion lymph node dissection will hopefully address the utility of this strategy in patients with a positive sentinel lymph node. While the optimal follow up for patients who elect not to undergo any lymph node assessment remains undefined, the high rate of nodal relapse in this study suggests that this may be a reasonable option for this patient population as well. As the risk of recurrence peaked in the first two years in this study, intensive screening with radiologic studies and frequent physical exams beyond this point may be less useful, as relatively fewer recurrences would be detected. This study represents an initial step in creating a framework for future prospective evaluation of surveillance strategies by identifying high risk periods for recurrence. In the current era of resource management, a data driven method of surveillance for melanoma patients, as well as the determination of the most common sites of recurrence may allow for a defined surveillance strategy that is both cost effective and patient focused.
